# A Model of Discovery: The Role of Imaging Established and Emerging Non-mammalian Models in Neuroscience

**DOI:** 10.3389/fnmol.2022.867010

**Published:** 2022-04-14

**Authors:** Elizabeth M. Haynes, Tyler K. Ulland, Kevin W. Eliceiri

**Affiliations:** ^1^Morgridge Institute for Research, Madison, WI, United States; ^2^Center for Quantitative Cell Imaging, University of Wisconsin-Madison, Madison, WI, United States; ^3^Department of Pathology, University of Wisconsin-Madison, Madison, WI, United States; ^4^Department of Biomedical Engineering, University of Wisconsin-Madison, Madison, WI, United States; ^5^Department of Medical Physics, University of Wisconsin-Madison, Madison, WI, United States

**Keywords:** microscopy, model organisms, neurodegeneration, zebrafish, zebra finch, *Danionella*, emerging model organisms, intravital imaging

## Abstract

Rodents have been the dominant animal models in neurobiology and neurological disease research over the past 60 years. The prevalent use of rats and mice in neuroscience research has been driven by several key attributes including their organ physiology being more similar to humans, the availability of a broad variety of behavioral tests and genetic tools, and widely accessible reagents. However, despite the many advances in understanding neurobiology that have been achieved using rodent models, there remain key limitations in the questions that can be addressed in these and other mammalian models. In particular, *in vivo* imaging in mammals at the cell-resolution level remains technically difficult and demands large investments in time and cost. The simpler nervous systems of many non-mammalian models allow for precise mapping of circuits and even the whole brain with impressive subcellular resolution. The types of non-mammalian neuroscience models available spans vertebrates and non-vertebrates, so that an appropriate model for most cell biological questions in neurodegenerative disease likely exists. A push to diversify the models used in neuroscience research could help address current gaps in knowledge, complement existing rodent-based bodies of work, and bring new insight into our understanding of human disease. Moreover, there are inherent aspects of many non-mammalian models such as lifespan and tissue transparency that can make them specifically advantageous for neuroscience studies. Crispr/Cas9 gene editing and decreased cost of genome sequencing combined with advances in optical microscopy enhances the utility of new animal models to address specific questions. This review seeks to synthesize current knowledge of established and emerging non-mammalian model organisms with advances in cellular-resolution *in vivo* imaging techniques to suggest new approaches to understand neurodegeneration and neurobiological processes. We will summarize current tools and *in vivo* imaging approaches at the single cell scale that could help lead to increased consideration of non-mammalian models in neuroscience research.

## Introduction

### The Historical Combined Power of Observation and Animal Models in Neuroscience

The earliest roots of modern neuroscience were born out of careful observation of neurons in animal models. Ramón y Cajal used light microscopy and sparse neuronal labeling in various mammalian and non-mammalian animal models to identify structures like growth cones and dendritic spines and propose theories of how the brain functioned. The power of basic observation combined with light microscopy and animal models has not become less important since: experiments in chicken embryos and frogs were essential to our early understanding of the rules that governed neurodevelopment (Hamburger, 1934; Levi-Montalcini and Levi, 1943; Gaze, 1959; Erdogan et al., 2016), *Caenorhabditis elegans* gave us the first connectome of an organism (White et al., 1986), and the use of fruit flies, chicken embryos, and rodents helped improve our understanding of the genetic pathways and ligands that control axon guidance (Letourneau, 1975; Seeger et al., 1993; Halloran and Kalil, 1994; Tessier-Lavigne, 1994; Polleux et al., 1998). The diffraction limited resolution of light microscopy initially constrained the study of ultrastructure of the neuron–the organization of organelles and cytoskeletal structures–to be accessible only through electron microscopy (Palay, 1954). However, fluorescence labeling and eventually super-resolution microscopy advanced the study of the neuronal cytoskeleton and its supporting organelles by enabling cell-level imaging with temporal resolution that is not possible with electron microscopy. The use of fluorescence light microscopy also allows for individual proteins and structures to be followed over time at a relatively high spatial (sub-cellular) resolution. This allowed microscopy data to rapidly move away from being solely pictures illustrating observed phenomena, and instead become quantifiable data that can reveal biological changes with mechanistic significance.

The close relationship between microscopy and neuroscience has not been one-sided: the use of animal models to study the function and anatomy of the nervous system has also been a tremendous technology driver for the development of new imaging methods and reagents. There are continuous efforts in the bioimaging community for developing optical imaging technologies capable of imaging faster at a higher resolution and deeper into tissues. To prove the effectiveness of new imaging tools or analysis methods, test models are needed. While *ex vivo* imaging phantoms have utility in testing new methods, *in vivo* imaging, especially of the brain, is often used as a gold standard for the success of a new optical imaging technology. For example, after the development of the modern laser scanning confocal in 1986 (Amos and White, 2003), a range of specimens including chick, *C. elegans* and *Drosophila* were used to demonstrate its ability to optically section complex samples (White et al., 1987). The advent of the laser scanning confocal was followed by 2-photon microscopy in order to overcome the problems of scattering and diffraction that hindered deep tissue imaging (Denk et al., 1990). 2-photon was promptly applied to image neurons in brain slices (Yuste and Denk, 1995; Svoboda et al., 1996), and expression of green fluorescent protein (GFP) in the brain of larval Drosophila (Potter et al., 1996).

The use of small, accessible models to benchmark new technology demonstrates another important concept: cutting edge technology should be paired with the most compatible samples to fully leverage its potential. While imaging in human tissues has been critical in the past for understanding neurodegenerative disease pathology, primary human tissue cannot be genetically manipulated and is generally not compatible with live optical imaging, reducing the utility of most advanced microscopy methods. Additionally, most available human tissue often reflects advanced stages of disease, making it difficult to understand the early processes driving neurodegeneration. While brain organoids are easier to image and can be manipulated in more ways, they lack the physiological context and organization of brain tissue. To comprehensively dissect mechanisms of brain function and disease it is necessary to use intravital imaging and animal models (Bellen et al., 2010; Dawson et al., 2018; Fontana et al., 2018; Rapti, 2020).

### Opportunities and Challenges of *in vivo* Observation

**FIGURE 1:**
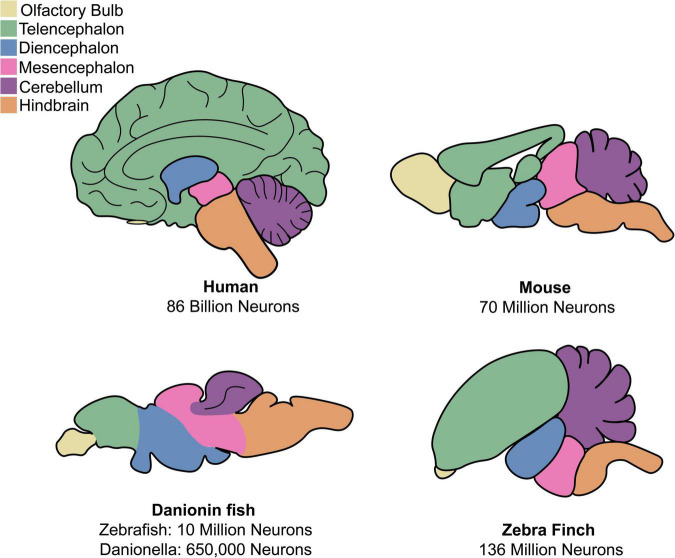
Comparison of anatomy for mammalian and discussed non-mammalian vertebrates. Both mammalian and non-mammalian vertebrates share similar brain anatomy, though region size and organization varies greatly between species.

Human and non-human primates have large and complex brains, ranging in size from the ∼86 billion neurons of humans to the 634 million neurons of the marmoset (Herculano-Houzel, 2009). An advantage of non-primate models are their more compact nervous systems, which are easier to manipulate and image. *C. elegans* famously has only 302 total neurons (White et al., 1986), making early attempts at circuit-tracing possible. Comparatively, the brain of *Drosophila* has ∼100,000 neurons (Scheffer et al., 2020), an adult zebrafish brain has ∼10 million neurons (Hinsch and Zupanc, 2007), and a mouse brain has ∼70 million neurons (Herculano-Houzel et al., 2006). A drawback to the use of invertebrates are the anatomical and cellular dissimilarities between invertebrate nervous systems and human nervous systems. This can make it difficult to study diseases affecting specific areas of the human brain, as many neurodegenerative diseases do. Compared to invertebrates, brain anatomy and function are generally similar across vertebrates and comparable to humans, even in lower vertebrates like fish (Figure 1). Ideally, a scientist should select the least complex model organism that possesses all components necessary to model the aspect(s) of a disease they are interested in.

In many cases, the least complex animal best capable of answering fundamental questions on neurodegenerative disease is still a vertebrate. However, imaging the brain and nervous system in vertebrate animals presents unique challenges for optical imaging: (1) The brain is dense and hidden away behind a skull and skin that promotes scattering, (2) Individual neurons are small, with structures like synapses requiring super-resolution techniques to resolve them, (3) But axons and dendrites form complex architectures that span large areas. Addressing these challenges for mammalian models such as non-human primates and rodents commonly requires extracting and manipulating tissues–sectioning brains, culturing neurons, or clearing whole tissues. To image neurons *in vivo* in mammals, an approach known as intravital microscopy, surgery must be performed to increase access to the brain. A cranial window can be installed, where a portion of skull is removed and replaced with a coverslip (Levasseur et al., 1975; Svoboda et al., 1997; Cramer et al., 2021). Cranial windows have allowed for unprecedented intravital imaging in rodent and non-human primate (NHP) models but can also drastically change the local environment where they are installed. Cranial windows can induce inflammatory responses, alter cortical activity and hemodynamics, and result in temperature fluctuations at the site of the window (Bacskai et al., 2001; Kalmbach and Waters, 2012; Guo et al., 2017; Park et al., 2019; Cramer et al., 2021). Thinning of the skull is less invasive and can be preferable to installation of a cranial window but is technically difficult and can require periodic re-thinning over repeated imaging sessions (Christie et al., 2001; Cramer et al., 2021). Despite improvements in cranial window technology and technique, intravital imaging in mammalian models still requires compromise: lower resolution, smaller fields of view, shorter imaging periods and increased costs overall. Due to the difficulty in achieving high temporal and spatial resolution in intravital imaging in mammals, studies often focus on lower-resolution types of functional imaging (such as use of calcium sensors), or information about neuron density or morphology.

Cellular and sub-cellular phenomena that require very high temporal and spatial resolution to observe are largely outside the reach of intravital imaging. However, these cell-level dynamics are exceedingly important to understanding neuronal health and function. This includes organelle distribution, cytoskeletal organization, movement of cargos by molecular motors, interactions between neurons and glia, and metabolic state (Dubey et al., 2015; Sweeney et al., 2018; Borst et al., 2019; Qin et al., 2021; Wilson and Metzakopian, 2021). A push for broader use of non-mammalian models can help to bridge current gaps in spatial and temporal sampling, such as imaging at the single-cell level, where imaging is impossible or impractical in mammalian models.

Non-mammalian models can be selected for advantages like small size and accessible nervous systems, as well as useful properties such as increased optical clarity that make *in vivo* imaging easier and less invasive (Table 1). Non-mammalian models are also less expensive to use, and their smaller size makes it easier to cover large fields of view faster. Their short life cycles and quick time to maturity makes generation of mutant or transgenic animals faster and can speed up research studies. Lastly without the need for invasive surgery, use of non-mammalian vertebrates also faces lower regulatory barriers.

**TABLE 1:** Comparative summary of a selection of common and emerging vertebrate models.

Species	Human Tissue (*Homo sapiens*)	Macaque (*Macaca mulatta*)	Mouse (*Mus musculus*)	Zebra Finch (*Taeniopygia guttata*)	Zebrafish (*Danio rerio*)	Danionella (*Danionella cerebrum*)	Killifish (*Nothobranchius furzeri*)	Western Clawed Frog (*Xenopus tropicalis*)	Axolotl (*Ambystoma mexicanus*)
Lifespan	70-100 years	25-40 years	2-3 years	5-7 years	3-4 years	3-4 years	4-8 months	n/a, likely similar to X. laevis (15-20 years)	10-15 years
Ages Studied	adult-advanced age	adult	embryo-adult	adult	embryo-adult	embryo-adult	adult	embryo-adult	embryo-adult
Neurons in Adult	86 billion (Herculano-Houzel, 2009)	6.4 billion (Herculano-Houzel, 2009)	70 million (Herculano-Houzel et al., 2006)	136 million (Olkowicz et al., 2016)	10 million (Hinsch and Zupanc, 2007)	650,000	n/a	n/a	n/a
Skull thickness	6.5-8mm depending on area (Li et al., 2007)	2-4mm depending on area (Nie et al., 2011)	310-340μm (Soleimanzad et al., 2017)	~300μm (Abi-Haidar and Olivier, 2009)	~20-25μm (Castranova et al., 2021)	no skull roof	n/a	n/a	n/a
Ex-vivo cultures	uncommon	uncommon	yes	yes	yes	no	no	yes	yes
									
Live Optical Imaging	**−**	**+**	**+**	**+**	**++**	**+++**	**−**	**−**	**−**
									
Genetics	**−**	**+**	**+++**	**+**	**+++**	**++**	**++**	**+**	**+**
									
Behavior	−	Broad array of behaviors including social, learning, memory, and exploration.	Broad array of behaviors including social, learning, memory, and exploration.	Most notable for vocal learning and sexual dimorphism in vocal behavior.	Broad array of behaviors in adults and larvae, including predator avoidance, social behavior, learning, memory, and exploration.	Broad array of behaviors in adults, including vocalization, social behavior, and learning, memory and exploration.	Broad array of behaviors in adults, including social behavior, learning, memory, and exploration.	Adult vocalization and tadpole swimming have been studied.	No reported studies of stereotyped behavior.
Regenerative	**−**	**−**	**−**	**+**	**++**	not reported, but likely	**++**	**+**	**+++**
									
Useful Features	High relevance to disease. Necessary for initial characterization of pathologies and genes associated with a disease.	Primate with ~92% genetic conservation to humans and high physiological relevance to human disease.	Mammal with high physiological relevance to human disease. Highly established protocols.	More closely models human vocalization and vocal learning than other available models.	Small vertebrate, transparent in the embryonic and larval stages. Pigmentless mutants available for adults. Well established model with many resources.	Small transparent vertebrate with the smallest known brain of any vertebrate. Amenable to genetic techniques used in most teleost fish.	Short-lived fish that displays aging-associated cognitive defects, neurodegeneration and muscular degeneration. Embryos can suspend aging during “diapause” for months to years.	Smaller Xenopus species with a compact diploid genome that reaches maturity faster than X. laevis. Useful for cell and developmental biology and genetics.	Extreme ability to regenerate brain and peripheral nervous system.
Drawbacks	Limited tissue availability, no temporal context available, usually limited to histopathology.	Expensive, prolonged time to maturity. Difficult to image due to skull, skin, and fur. Ethical concerns.	Generation of mutants takes a long time. Does not recapitulate all human disease phenotypes. Difficult to image due to skull, skin, and fur.	There is no reliable way to generate transgenic animals.	Limited working antibodies. Genetic duplications in teleosts can make gene editing challenging.	Produces relatively few embryos. Neotenic traits may complicate use as disease model.	Few resources currently available. Male-male aggression complicates housing.	Pigmented tissue makes imaging impractical, though albino strains exist and mutants lacking yellow pigment have been made (Nakajima et al., 2021).	Excessively large genome size, no true pigmentless mutant, neotenic traits may complicate use as disease model

*Signs denote ease or ability to use the model for a particular purpose. -, difficult or not possible; +, possible; ++, easily possible; +++, very easy, model is well suited to this purpose.*

The power of optical imaging technologies like 2-photon (Denk et al., 1990) and light sheet microscopy (LSFM) (Huisken et al., 2004) have brought revolutionary improvements to our ability to observe cellular level dynamics *in vivo*, while specialized microenvironment sensitive imaging techniques such as fluorescent lifetime imaging (FLIM) and use of biosensors (Leopold et al., 2019; Datta et al., 2020) can tell us more about dynamics within *in vivo* sub-cellular domains. With the notable exception of LSFM, many advanced imaging techniques have been under-utilized in non-mammalian models. What would it take to expand the repertoire of advanced imaging techniques in non-mammalian vertebrates?

### A New Age for Non-mammalian Models

Approximately 45% of neurobiology papers published in the last decade have relied on the use of rat and mouse models, compared to 2% for NHP and 6% for other common vertebrate and non-vertebrate models.^1^ There are many reasons for the extensive use of rodents in the field, mostly related to the availability of tools and reagents, sequenced genome, and physiological similarities to humans. It is unquestionable that rodent models represent many important aspects of human disease, but what biological opportunities do researchers leave on the table by optimizing only the technological approaches without addressing their choice of animal model as well? Given the rapid expansion of genomic, transcriptomic, and microscopy tools available to the average neurobiologist, it is possible to select models for biological qualities that help address specific neurobiological questions, instead of for their generalist versatility.

Calls for the diversification of model organisms in neurobiological research are hardly new (Manger et al., 2008; Keifer and Summers, 2016; Russell et al., 2017; Yartsev, 2017; Żakowski, 2020). The arguments for introducing new model systems can vary depending on need: for some, it is necessary to find animals that mirror aspects of complex human behavior such as vocal learning or social dynamics, for others, it is enough for an animal to have some special characteristic that allows for new experimental flexibility. *C. elegans* was initially proposed for connectome mapping by 2002 Nobel laureate Sydney Brenner in part because the *Drosophila* brain was too large for the project (Emmons, 2015). Serendipitously, 2008 Nobel laureate Martin Chalfie credits his work with Brenner and *C. elegans* for his decision to utilize GFP to visualize biological structures: “[…] if I had not worked on *C. elegans* and constantly told people that one of its advantages was that it was transparent, I am convinced I would have ignored GFP when I first heard of it” (Chalfie, 2009). The use of diverse model organisms can inform new scientific thinking and inspire innovation.

To best understand how to encourage re-evaluation of model choice and awareness of new model systems for neuroscience research, we must first ask what has contributed to the lack of diversity in the animal models widely used for neuroscience research. One key factor is that initially there were limited ways in which to manipulate animals biologically, and so scientists had to rely on what biology was available to study through natural diversity. As powerful new tools for genetic manipulation were developed, the field began to condense into a handful of models that had sequenced genomes, and well-defined protocols for housing, breeding, and experimental manipulation. This has generally been a boon for research, enabling researchers across the world to speak in a shared language of standardized experimental approaches and reagents. Today, though, it is easier than ever to sequence and edit an organism’s genome. CRISPR has proven to be easily adaptable to many different model systems, and there is a plethora of tools for next generation sequencing and transcriptomics. The pace at which reagents, protocols, and knowledge can be disseminated to create a “shared language” within a field has also increased, fueled by initiatives like protocol-focused websites and journals, preprints, social media, and organism-dedicated societies.

We therefore propose that any animal can become a useful model for neurobiological disease research if it meets the following conditions: (1) The model represents a useful structure, system, or behavior related to neurobiology, (2) It is easily acquired, maintained, and bred in a lab environment, (3) It can be genetically manipulated, and (4) There is a way to measure perturbations to the nervous system through behavioral output, imaging, or other measurements. For the purposes of this review, we will focus on three rising non-mammalian vertebrates–Zebrafish (*Danio rerio*), *Danionella* species, and zebra finch (*Taeniopygia guttata*)–that are both amenable to cutting-edge optical imaging techniques at a cellular-level resolution and well-suited to serve as models of neurodegenerative disease. We will also discuss the cellular targets that would benefit from study in non-mammalian vertebrates. We will briefly describe some of the advanced imaging and cell labeling techniques that have been successfully used to look at neurobiological disease in non-mammalian models, give an overview of some of the current gaps in tools and knowledge, and discuss additional emerging models and ways in which the field could expand and grow.

### Targets of Imaging at Cellular and Sub-Cellular Resolution *in vivo*

Emerging questions in neurodegenerative disease are increasingly focused on the interplay of multiple systems within the brain during disease progression–and microscopy is perfectly suited to interrogate how multiple cellular systems interact in a temporal and spatial manner. Fluorescence labeling and endogenous signals (such as second harmonic generation of fibrillar collagen and intrinsic metabolic fluorescence) allow tracking of several structures or cell types simultaneously in living animals. Using imaging techniques with high temporal and spatial resolution, we can also assess cells not as a single homogenous unit, but as dynamic structures with regions that react and respond to their local environment. In addition, the use of genetically encoded biosensors can inform us of how cell-cell or cell-environment interactions can change intracellular calcium, membrane voltage, redox potential, and more (Yano et al., 2010; Bando et al., 2019; Zhang Y. et al., 2021).

Some of the major features that can be examined through imaging are the contribution of the “neurovascular unit” (NVU) and the brain’s immune cells, microglia, in neurodegenerative disease. The NVU is defined as the multiple interacting elements, both cellular and extracellular, that regulate blood flow and blood brain barrier function. This includes not only the neurons and the vasculature itself, but also the microglia, pericytes, and astrocytes that provide support and help maintain cerebral homeostasis (Iadecola, 2017; Sweeney et al., 2018; Schaeffer and Iadecola, 2021). Though previously the study of neurodegeneration and cerebrovascular disease were considered distinct, the theory of the NVU has been a fruitful subject of research since its conception (Iadecola, 2017). Defects in cerebrovascular blood flow have been found in Alzheimer’s disease (AD) and other dementias, Huntington’s disease (HD), Parkinson’s disease (PD), amyotrophic lateral sclerosis, and other neurodegenerative diseases (Rule et al., 2010; Melzer et al., 2011; Chen J. J. et al., 2012; Murphy et al., 2012; van de Haar et al., 2016; Leeuwis et al., 2017; Leijenaar et al., 2017; Sweeney et al., 2018).

Microglia, resident phagocytic glia in the brain, have been an implicated in the pathogenesis of Alzheimer’s disease and other neurodegenerative diseases (Srinivasan et al., 2016; Wang et al., 2016; Füger et al., 2017; Huang et al., 2017; Masuda et al., 2019; Prinz et al., 2019; Tejera and Heneka, 2019). Microglia are not only responsible for clearing debris and pathogens from the central nervous system (CNS), but also function in synaptic remodeling and secrete signaling factors that contribute to immune response and tissue repair (Davalos et al., 2005; Nimmerjahn et al., 2005; Paolicelli et al., 2011; Schafer et al., 2012; Miyamoto et al., 2016; Weinhard et al., 2018; Davies et al., 2019). Like macrophages, they are dynamic and migratory cells that have different morphological phenotypes and close interactions with other cells in the brain. Studying microglia requires the ability to image a wide field of view (due to their migratory nature), deep into tissue at a high temporal and spatial resolution which is difficult to achieve in larger mammalian brains. Microglia are present in both mammalian and non-mammalian vertebrates, and microglia-like cells have been extensively described in invertebrate annelids (Sharma et al., 2021). The existence of distinct microglia in other invertebrates, such as *Drosophila*, is less clear and suggests they may have arisen convergently in annelids (Hartenstein and Giangrande, 2018). Study of microglia in small non-mammalian vertebrates, and comparative study of annelid and vertebrate microglia could improve our overall understanding of microglia function in CNS homeostasis and disease.

Astrocytes are another glial component of the NVU that mediate connections between neurons and the vasculature to provide synaptic support and control cerebral blood flow (Braun and Iliff, 2020). Astrocytes accomplish this through physically contacting synapses and vasculature, forming an incredibly complex morphology with up to 80% of the cell’s membrane being used in fine processes (Ventura and Harris, 1999; Ogata and Kosaka, 2002; Mathiisen et al., 2010; Reeves et al., 2011; Khakh and Sofroniew, 2015). The complex morphology of astrocytes, alongside their complex role in connecting multiple cell types, makes them excellent candidates for microscopic study with increased spatial and temporal resolution.

Underlying the cellular-level phenomenon we can observe are the sub-micron structures that facilitate cellular behavior. The neuronal cytoskeleton drives neuronal development but must also be maintained throughout the life of an organism. Defects in the neuronal cytoskeleton can cause cell death through signaling cascades as well as failure in cargo trafficking (Dubey et al., 2015). Mitochondria produce the ATP necessary for cells to function, but also play a role in calcium buffering and generation of reactive oxygen species. Multiple neurodegenerative diseases are known to induce defects in the neuronal cytoskeleton and mitochondria (Dubey et al., 2015; Mandal et al., 2018), but the relationship between this damage, disease progression, and neuronal death are still unclear. Additionally, other sub-micron structures such as endoplasmic reticulum-mitochondria contact sites, endosomes, and lysosomes are relevant for the study of neuronal function and disease (Mandal et al., 2018; Peng et al., 2019; Wilson and Metzakopian, 2021). Imaging of these structures is technically challenging and typically requires targeting superficial neurons and sparse labeling techniques, coupled to sensitive, high-resolution microscopes capable of rapid acquisition speeds.

### Neuroimaging Tools for *in vivo* Applications

There have been multiple comprehensive reviews recently published on microscopy techniques in neuroscience (Choquet et al., 2021; Werner et al., 2021) and on LSFM specifically (Power and Huisken, 2017; Fiolka, 2019; Hillman et al., 2019; Lemon and McDole, 2020; Stelzer et al., 2021). Therefore, these techniques will only be reviewed in brief in the context of their usefulness for addressing problems in neurodegenerative disease.

#### Confocal Imaging

Laser-scanning and spinning disk confocal imaging are among the most commonly used techniques in neurobiological imaging (Tanaami et al., 2002; Amos and White, 2003). Confocal microscopes can be in upright or inverted configuration and are suited for use with traditional slides and coverslips. Confocal microscopy generates optical sectioning by its use of a pinhole to reject light from outside the desired focal plane. The rejection of light produces high signal to noise (SNR) imaging, however, exposure of the sample to laser light is still a viability concern. Since regions of the sample outside of the plane of focus are still in the cone of illumination, excess light is delivered to the sample and living samples are at risk of increased phototoxicity and photobleaching. Scanning confocal microscopes are also typically slow, making them best suited to fixed samples or live samples that do not require high temporal resolution views.

Spinning-disk confocal microscopes trade a fixed pinhole (single point scanning) for an array of pinholes on a mechanically spinning disk (multi-point scanning). These pinhole arrays allow for parallel illumination of the sample. Parallelization of illumination and detection reduces the amount of light the sample is exposed to and increases the speed of imaging. This makes spinning-disk confocal typically more suitable than scanning confocal for fast *in vivo* imaging of cellular and sub-cellular phenomenon (Fuseler et al., 2018).

Additional gains in scan speed can be achieved through swept field confocal technology and similar approaches (Castellano-Muñoz et al., 2012). In this technique, the mechanical spinning disk is jettisoned in favor of a fixed aperture plate. Light from the aperture plate is “swept” over the sample by galvanometric and piezo-mirrors which can reach faster scan speeds than spinning disk or laser scanning technology. The high speeds of swept field confocal are useful for imaging cellular and sub-cellular dynamics *in vivo* (Castellano-Muñoz et al., 2012).

All confocal techniques suffer difficulties with penetration depth in complex animal tissues due to scattering and diffraction of light, and so are best suited to imaging of flattened tissues or superficial structures. Confocal microscopes are generally used with laser lines between 400 and 600 nm.

#### 2-Photon and 3-Photon Microscopy

Multiphoton microscopy, which includes 2- and 3-photon microscopy, relies on the principle that a fluorophore can be excited by 1-photon at its exact excitation wavelength, but it can also be excited by two or three simultaneous (<1 femtosecond apart) photons which are two to three times the wavelength of its normal excitation, respectively. For example, a fluorophore could be excited with one photon at 488 nm, two photons at 976 nm, or three photons at 1464 nm. Both 2-photon and 3-photon microscopy benefit from the deeper penetration afforded by longer wavelengths of light, and thus are the choice for many *in vivo* neuroimaging applications (Denk and Svoboda, 1997; So et al., 2000). 2-photon microscopes use lasers in the 700–1000 nm wavelengths, while 3-photon microscopes tend to use lasers between 1300 and 1700 nm wavelengths. In addition to depth, the long wavelength used for excitation can provide better viability when compared to traditional brightfield or confocal microscopy. 3-photon microscopy can be hindered by the currently limited range of fluorophores that are capable of being excited by its longer infrared wavelengths.

#### Light Sheet Fluorescence Microscopy

Light sheet fluorescence microscopy, also known as selective plane illumination microscopy (SPIM), is a technique in which a cylindrical lens is used to shape a laser beam into a thin gaussian sheet of light (Huisken et al., 2004). The light sheet constrains illumination of the sample only on the focal plane of interest, allowing optical sectioning without the need to reject any out of focus light. This avoids delivery of excess laser light to the sample which reduces photobleaching and phototoxicity. In addition, the use of high-speed scientific cameras reduces the time it takes to acquire a volume, further reducing laser exposure and enabling high-volume, long-term imaging while limiting damage to the sample. LSFM systems are generally highly adaptable and can be built around the requirements of the sample, making them well-suited to a diverse range of model systems (Power and Huisken, 2017).

Light sheet fluorescence microscopy tends to be hindered by tissue-induced scattering, which deforms the light sheet itself and causes striping and deterioration in the resulting image. This can be combatted by using multi-view systems that allow for illumination and imaging from two sides of the sample (Huisken and Stainier, 2007; Krzic et al., 2012). A final image can be reconstructed by piecing together the two halves of the image. This increases the time required for acquisition and the amount of light the sample is exposed to, so it is not a universal solution.

Alternative beam strategies for LSFM have also been developed, notably 2-photon, Bessel beam, lattice, and Airy light sheets, as well as the recent field synthesis method (Planchon et al., 2011; Truong et al., 2011; Chen et al., 2014; Vettenburg et al., 2014; Liu et al., 2018; Chang et al., 2019). These alternative beams combat the diffraction and scattering of a traditional light sheet and can create thinner and more uniform light sheets which improve resolution and image quality.

#### Single Objective Light Sheet Microscopy

Traditional LSFM set-ups utilize two objectives oriented at an orthogonal angle to each other, resulting in spatial constraints regarding the size and angle of the objective’s nose cone. This limited LSFM to lower numerical aperture (NA) lenses until single-objective LSFM solutions were introduced (Botcherby et al., 2007; Dunsby, 2008; Bouchard et al., 2015; Fadero et al., 2018; Kim et al., 2019; Yang et al., 2019; Sapoznik et al., 2020). The continued evolution of single-objective light sheet systems could lead to the wide-spread adoption of these microscopes for high-speed, long-term *in vivo* cellular and sub-cellular imaging.

#### Super-Resolution Techniques

Limited super-resolution techniques have been applied to neurons *in vivo.* Stimulated emission depletion microscopy (STED) has been used to image dendritic spines and even synaptic structures in a living mouse (Ter Veer et al., 2017; Wegner et al., 2017, 2018; Masch et al., 2018). Single-molecule STORM using an oblique plane strategy was introduced in 2019, allowing for tissue-scale super-resolution imaging in Drosophila and stickleback fish (Kim et al., 2019). Though limited to fixed tissues, cleared tissue and expansion microscopy techniques also allow for high-resolution tissue-scale imaging (Chen et al., 2015; Wassie et al., 2019; Porter and Morton, 2020; Weiss et al., 2021).

#### “Smart” Microscopy and Image Analysis

Smart, adaptive microscopy techniques alongside of other advanced image processing routines such as deconvolution and machine learning can yield additional insights from collected data and reduce the burden of large datasets through improved visualization and automation. This is critically important for brain imaging, where computational solutions are needed for challenges including low signal-to-noise ratio, areas of divergent image quality, and extremely fast recording times (as in imaging of calcium dynamics or voltage sensors). Smart microscopy, where images are computational analyzed in real-time, has been successfully applied in LSFM to automatically rotate the sample for improved imaging based on detected image quality (He and Huisken, 2020). Adaptive focus imaging has also been applied alongside light sheet imaging to keep embryos in focus despite dramatic changes in shape over development (Royer et al., 2016, 2018; Ji, 2017; McDole et al., 2018). Improvements in image quality for lower-resolution or lower signal-to-noise ration images can be obtained through deconvolution and denoising tools. Deconvolution is a relatively standard technique for fluorescent microscopy, but new strategies for deconvolution such as super-resolution radial fluctuations (SRRF) are being developed (Gustafsson et al., 2016; Culley et al., 2018). Deconvolution can also be coupled to machine learning to improve the resolution of low-quality images by training a neural network on higher quality images (Weigert et al., 2018; Krull et al., 2019). Machine learning techniques have been widely used in modern fluorescent microscopy for denoising and image quality improvement as well as segmentation of images. There are many open source machine learning tools available that give biologists the option to train deep-learning models based on their own datasets, or utilize publicly accessible pre-trained models (Moen et al., 2019; Weigert et al., 2020; Stringer et al., 2021; von Chamier et al., 2021). The importance of such open access computational tools, including widely used open source image processing platforms such as ImageJ (Schindelin et al., 2012), will keep growing as the limits of light microscopy continue to be pushed. Advances in computational imaging have great opportunity to enable new capabilities in established and emerging animal models.

### Rising Non-mammalian Vertebrate Models for the Study of Neurodegenerative Disease

#### Zebrafish

##### Background and Genetic Tools

Danio rerio, the zebrafish, has been a rising star within neuroscience since it was championed by George Streisinger starting in the 1970s (Varga, 2018). Though it was just one of many bony fish models used at the time, it has left the world of “emerging model organisms” to become an example of successful widespread adoption of a “new” model. The optical clarity of zebrafish during embryonic and larval stages offers unprecedented access to imaging for a vertebrate, and its reduced size means that imaging whole brain volumes is increasingly achievable. Their potential as a disease model is good, with 82% of human disease genes have a zebrafish ortholog (Howe et al., 2013). Zebrafish spawn in large clutches of ∼100 embryos, making them popular for large scale pharmacological, genetic, and behavioral screening. To this end, multiple well-established methods exist for genetic manipulation in zebrafish, including CRISPR/cas9 editing and transgenesis *via* Tol2 mediated insertion (Kawakami, 2005; Kwan et al., 2007; Don et al., 2017; Liu et al., 2019). However, it is worth noting that zebrafish have ∼30% of their genome duplicated, which can complicate the creation of mutant or knockout animals as multiple genes may need to be targeted (Woods et al., 2000, 2005; Howe et al., 2013). Existing zebrafish mutants and transgenic lines can be acquired easily through an international repository (ZIRC), and a large library of phenotypically interesting mutants already exist thanks to an important large scale ENU mutagenesis effort (Driever et al., 1996).

Aiding in their utility as a genetic model, zebrafish are quick to develop. They reach juvenile status at 30 days post-fertilization and become sexually mature adults at ∼90 days post-fertilization. Mice, in comparison, reach sexual maturity at ∼55 days post-fertilization, making them slightly faster to mature but relatively comparable. Zebrafish are generally kept for 2 years in lab housing facilities but can survive for as long as 5 years. Their lifespan along with detectable aging-associated changes make them a model with great potential for addressing neurodegenerative and aging-associated diseases (Keller and Murtha, 2004; Kishi, 2004; Adams and Kafaligonul, 2018).

Zebrafish have already proven useful models for neuroscience because of their small and accessible brains. The structure and composition of the zebrafish nervous system shares much in common with that of humans, including a general architecture of a spinal cord, hindbrain, midbrain, and forebrain and predominantly similar neurochemical components (Figure 1; Kimmel, 1993; Bae et al., 2009; Panula et al., 2010). One notable anatomical difference between mammalian and teleost fish brain structure is that fish lack a cerebral cortex. All mammalian CNS glial cell types are also found in zebrafish, namely, oligodendrocytes, astrocytes, and microglia (Kawai et al., 2001; Yoshida and Macklin, 2005; Avila et al., 2007; Lyons and Talbot, 2014; Xu et al., 2015; Chen et al., 2020).

In addition to brains that are largely comparable to mammals, adult zebrafish also have a host of complex behaviors that parallel many established murine tests of learning and memory, anxiety, and social preference (Orger and de Polavieja, 2017). The similarities between zebrafish and mouse behavioral assays allow comparisons to be made between the substantial body of literature in mice and new work in zebrafish.

##### Imaging Neuronal Function During Behavior

Pairing behavioral assays with live imaging has immense potential for the study of neurological disease, especially neurodegenerative diseases that are known to cause motor, learning, and memory deficits. These kinds of experiments are increasingly common, though technical challenges limit their widespread adoption. One such caveat is that imaging behaving larvae generally requires stabilization, either through mounting or paralysis through genetic or chemical means. This stabilization reduces much of the natural feedback received by the fish while enacting the behavior. Stabilized or partially stabilized larvae have been used with multiphoton and LSFM microscopy to image genetically encoded calcium indicators (GECIs) in opto-motor control, auditory and visual response, olfaction, and prey-capture decisions (Förster et al., 2020; Wang et al., 2020; Bruzzone et al., 2021; Herrera et al., 2021). The introduction of adaptive imaging techniques and real-time correction for movement allow for reduced levels of restraint while improving image quality (Wang et al., 2014; Rodríguez and Ji, 2018; Griffiths et al., 2020). This leaves the ability to image reaction to virtual stimuli, but without natural feedback from the environment or sensory systems such as the lateral line or vestibular system. Real-time virtual feedback stimulation can be provided in response to active behaving (Portugues and Engert, 2011; Ahrens et al., 2013; Trivedi and Bollmann, 2013; Bianco and Engert, 2015; Jouary et al., 2016; Kawashima et al., 2016; Naumann et al., 2016; Huang et al., 2020), but great interest still remains in functional neuronal imaging of freely swimming fish.

Currently, multiple solutions for imaging of freely behaving larvae exist, most with widefield fluorescence illumination and detection combined with methods to perform tracking and predictive adjustments of optical elements (Cong et al., 2017; Kim et al., 2017; Symvoulidis et al., 2017). 2-photon and LSFM solutions to image true freely behaving larvae are technically difficult to implement as they require precise direction of the illumination area. Despite their relative speed compared to other techniques, acquiring whole brain volumes using 2-photon or LSFM takes additional time compared to a single widefield acquisition, making it slow in comparison to the speed of a moving larva. Light field microscopy presents an alternative approach, providing speed at the sacrifice of spatial resolution (Levoy et al., 2006). Confocal light field microscopy has recently been implemented to improve resolution for volumetric imaging of neuronal GECIs in freely swimming larvae (Zhang Z. et al., 2021).

##### Imaging Cellular Dynamics

Both scanning and spinning disk confocal microscopy have been standard methods in the field for imaging cellular dynamics in development and disease in the zebrafish. Since its introduction in 2004, however, LSFM has revolutionized imaging cellular dynamics in embryonic and larval zebrafish, particularly during development. The use of mounting methods that preserve the 3D integrity of the sample is an important principle in LSFM, and zebrafish embryos can be mounted for LSFM imaging purely in water using FEP tubes and an agarose plug (Power and Huisken, 2017). The ability to mount and image without using a constrictive mounting medium allows for a much more natural progression of shape change and morphogenesis during development. This can be combined with adaptive imaging techniques to keep embryos in focus and within the field of view over many hours or days of development (McDole et al., 2018; Daetwyler et al., 2019; He and Huisken, 2020). Little work has been done specifically using LSFM to look at cell-scale phenomenon, like glia and the NVU in neurodegenerative disease in zebrafish. However, foundational work has been completed in characterizing lymphatics, vasculature, astrocytes, and microglia in the zebrafish brain using both LSFM and standard confocal microscopy.

Vascular imaging in zebrafish is a relatively mature field with established transgenic lines and existing protocols for the segmentation and quantification of complex vascular networks, including cerebellar vasculature (Chen Q. et al., 2012; Daetwyler et al., 2019; Kugler et al., 2020, 2021). A newly published zebrafish transgenic line marking astrocytes will prove a valuable resource to illuminating the role of astrocytes in development and disease (Chen et al., 2020) and could be combined with existing transgenic lines (Lawson and Weinstein, 2002) marking vasculature to better understand interplay between vascular systems and glia in the NVU.

Use of the zebrafish model has also enabled imaging of the glymphatic network of the CNS, a traditionally difficult imaging subject due to its complexity and location. Castranova et al. (2021) achieved live imaging of immune cells within meningeal lymphatics in larval and juvenile zebrafish brain. Zebrafish have thin skulls, and when coupled to a pigmentless strain they were able to image the meningeal lymphatics in intact animals through the skull using spinning disk confocal. This work shows the promise of non-invasive cellular-level imaging in the intact juvenile zebrafish, and positions zebrafish as a model to understand the interplay of the glymphatic network with neurodegenerative diseases.

Microglia in zebrafish are a rapidly expanding topic of interest–more papers have been published between 2019 and 2021 on microglia in zebrafish that in the preceding 12 years combined. Zebrafish microglia are generated in two waves, with a primitive wave derived from the primitive macrophages in the yolk sac, and a second wave that replaces the original population and originates from the hematopoietic stem cells of the dorsal aorta (Xu et al., 2015; Ferrero et al., 2018; Wu et al., 2020; Silva et al., 2021). Zebrafish microglia share a core transcriptional signature with mammalian microglia and are morphologically and functionally similar to microglia of other species (Oosterhof et al., 2017; Geirsdottir et al., 2019). Imaging studies in zebrafish have been used to show cross talk between neuronal activity and microglial morphology, phagocytosis of myelin sheaths and dying neurons, and response to long-range signaling cues (Peri and Nüsslein-Volhard, 2008; Li et al., 2012; Sieger et al., 2012; Mazaheri et al., 2014; Hughes and Appel, 2020). Two populations of zebrafish microglia have been observed: ameboid microglia, which have a higher capacity for phagocytosis, and the white-matter enriched ramified microglia, which actively remodel their protrusions and may play a more regulatory role (Wu et al., 2020). This is an interesting distinction because humans have heterogenous microglia populations, while other studied model organisms have been found to have more homogeneous microglia populations (Böttcher et al., 2019; Geirsdottir et al., 2019; Masuda et al., 2019; Sharma et al., 2021). If zebrafish microglia more closely resemble the heterogeneity of human microglia, they could fill an important niche in our understanding of microglia in human disease.

##### Sub-Cellular Dynamics

Embryonic and larval zebrafish have multiple subtypes of neuron with axons that grow superficially enough to allow for detailed imaging of sub-cellular structures at excellent resolution. The posterior lateral line (pLL) axons and the Rohon-Beard (RB) sensory neurons are two such commonly imaged populations. Imaging of microtubules (Stepanova et al., 2003; Asakawa and Kawakami, 2010; Lee et al., 2017; Haynes et al., 2021), mitochondria (Plucińska et al., 2012; Campbell et al., 2014; Paquet et al., 2014; Auer et al., 2015; Drerup et al., 2017; Mandal et al., 2018), ER-Mitochondria contact sites (Cieri et al., 2018; Vallese et al., 2020), endosomes (Clark et al., 2011; Ponomareva et al., 2014, 2016), and lysosomes (Drerup and Nechiporuk, 2013) is common in these superficial neurons in zebrafish embryos and larvae. When paired with genetic manipulation of disease-associated genes, zebrafish are an excellent tool to discern the molecular mechanisms behind basic disease processes.

Spinning disk and swept-field confocal microscopes have traditionally been the tool of choice to image fast-moving sub-cellular structures like microtubule comets and organelles. Zebrafish sensory axons, for example, can have axon arbors spread through a volume of 20–50 μm. That volume must be obtained in seconds or less to achieve adequate speed for measuring sub-cellular dynamics. However, progressive improvements in high NA light-sheet microscopes are enabling them to break into the sub-cellular niche. Lattice light sheet is difficult to implement but able to resolve objects of 200 nm, meaning that structures like Golgi and ER were able to be resolved in the brain of a living zebrafish embryo (Liu et al., 2018). Single objective light sheet as well as super-resolution techniques like STED have yet to be applied for sub-cellular imaging in zebrafish, however, the potential for their utility is high.

##### Disease Models

With an array of tools and techniques available for multiple ages, zebrafish are growing into their potential as an excellent model for neurodegenerative disease research. Disease-relevant mutations can be easily introduced through CRISPR/Cas9 editing or transgenic insertion, allowing the study of both the native function of the mutated gene as well as the disease-relevant consequences of its loss or mutation. Numerous disease models for neurodegenerative disorders including PD, AD, and hereditary spastic paraplegia (HSP) have been generated in zebrafish through genetic or non-genetic means (Fontana et al., 2018; Vaz et al., 2018; Naef et al., 2019; Wang and Cao, 2021).

Many of these diseases are complex and polygenic, making them difficult to model. For example, there are multiple genes associated with familial risk for PD, though most cases are spontaneous. In humans, PD results in neurodegeneration of the dopaminergic neurons and is accompanied by aggregation of the protein α-synuclein. PD can be modeled in the zebrafish through drug treatments that decrease dopamine levels or numbers of dopaminergic neurons or through genetic knockdown or knockout (Vaz et al., 2018). The role of synuclein genes and other PD associated genes have been studied in zebrafish and have been associated with defects in the dopaminergic system, mitochondrial dysfunction, and neurodegeneration (Milanese et al., 2012; O’Donnell et al., 2014; Vaz et al., 2018).

Alzheimer’s disease has been more difficult to model in zebrafish. A standard model of AD in mice is the 5xFAD mouse, which uses transgenic insertion of amyloid precursor protein (APP) and presenilin1 (PSEN1) with a constellation of 5 mutations associated with human familial AD (Oakley et al., 2006). An equivalent to this model has not been generated in zebrafish, though perturbation of presenilin-1 and −2 genes and zebrafish APP genes *appa* and *appb* have been widely used to test the role of these genes in development and disease (Kaiser et al., 2012; Luna et al., 2013; Fontana et al., 2018; Banote et al., 2020). Aβ peptide, the product of APP cleavage, has also been used directly in zebrafish to study the role of different length Aβ on biological processes and Aβ toxicity. Cerebroventricular injection of Aβ-42 showed AD-like phenotypes including neuronal cell death and microglial activation in both young and aged animals (Bhattarai et al., 2016, 2017). Microglia have been implicated in pathways controlling neuroproliferation in the zebrafish in response to Aβ injection (Bhattarai et al., 2020), though no live cell imaging of glia or neuronal cell interaction with plaques have been performed in zebrafish to date.

A strength of the zebrafish model is the ability to correlate phenotypes at the cellular and sub-cellular resolution with adult behavior and pathology. For example, a genetic zebrafish model of the progressive neurodegenerative disease hereditary spastic paraplegia showed defects in microtubule dynamics and axon stability during development as well as adult behavioral defects, demonstrating the ability to link early onset phenotypes to adult outcomes (Haynes et al., 2021). Further work to follow the consequences of genetic disruption of neurodegenerative disease risk genes from the sub-cellular level to specific circuits to whole-animal behavioral phenotypes could establish zebrafish as a revolutionary disease model.

#### Danionella

##### Background and Genetic Tools

*Danionella* are a genus of danionin fish species related to zebrafish, but smaller in size and with the peculiar advantage of being nearly completely transparent as adults. This remarkable characteristic is owed in part to their neoteny, or retention of juvenile traits. *Danionella* species range in length from 11 to 18 mm, which is roughly comparable to an older zebrafish larvae/a young juvenile (∼12 mm) (Conway et al., 2021). Many previously published papers on *Danionella* have stated the species used in their work is *Danionella translucida*. However, recently published morphological and molecular analysis showed that the widely used species in current research is *not D. translucida*, but a new species currently designated as *Danionella cerebrum* (Britz et al., 2021). Thus, we will use the designation *D. cerebrum* to discuss the species used in Penalva et al. (2018), Schulze et al. (2018), and Kadobianskyi et al. (2019). *Danionella cerebrum* possess the smallest adult vertebrate brain known, only eight times larger than a *Drosophila* brain (Schulze et al., 2018). Both *D. cerebrum* and *Danionella dracula* lack a bony roof on the top of the skull, making them highly amenable to brain imaging (Schulze et al., 2018; Conway et al., 2021). This allows the study of more established and mature circuits and brain regions with excellent resolution and relatively unobstructed field of view. Danionella cerebrum reach sexual maturity at 6–8 weeks, and spawn in clutches of around 12 eggs per female (Rajan et al., 2022). *Danionella* can be raised and maintained similarly to zebrafish, and their embryos develop at similar rates, making comparative studies between zebrafish larvae and *Danionella* adults straightforward, and avoiding additional investment in equipment (Penalva et al., 2018). Transgenesis in *Danionella* uses the same techniques applicable to zebrafish and other teleosts. Transgene insertion using Tol2 to express GCaMP6f and CRISPR/Cas9 mutagenesis of the *tyr* gene are both demonstrated successfully in Schulze et al. (2018). While the same tools for zebrafish transgene insertion can be used with *Danionella*, CRISPR gRNAs must be designed to be specific to available sequence for *Danionella* genes (Schulze et al., 2018; Kadobianskyi et al., 2019). Currently, no demonstration of genetic mutation of human-disease associated genes has been published.

##### Behavior

*Danionella* exhibit measurable social behavior and socially reinforced learning and have an additional surprising characteristic: males of both *D. translucida* and *D. dracula* species can vocalize. These vocalizations are short multi-pulse bursts and made primarily during displays of male-directed aggression (Penalva et al., 2018; Schulze et al., 2018; Tatarsky et al., 2021). Vocalization is not known to occur in zebrafish, so study of vocalization represents new possibilities in neurobiological research with this model. The ability to pair behavior with adult imaging, as discussed previously, is a huge strength of the fish systems presented here. Adult Danionella can be mounted and imaged in agarose as long as their gills remain exposed, unlike zebrafish larvae which do not yet depend on their gills for oxygenation (Schulze et al., 2018). This opens the possibility of live-imaging of neuronal calcium indicators in vocalizing fish presented with a visual cue of an intruding male, an exciting new way to assess behavioral circuits and sex differences in the brain (as females do not vocalize).

##### Sub-Cellular and Cellular Imaging

*Danionella* have immense potential for visualization of cellular and sub-cellular structures at high resolution within the brain of an adult animal. Though efforts have only begun recently, mosaic expression of GFP driven by the pan-neuronal promotor *elavl3* has been demonstrated to visualize sparsely labeled neurons in 5 days post-fertilization larval *D. cerebrum* using 2-photon microscopy. A region of the brain of a living adult *D. cerebrum* has been imaged from its dorsal surface to a depth of 376 μm, corresponding to the ventral surface (Penalva et al., 2018), and the brain vasculature of an adult *D. dracula* has been imaged to a depth of ∼800 μm using 2 and 3-photon microscopy (Akbari et al., 2021). These studies combined indicate that whole-brain live imaging is achievable in an adult vertebrate species.

The lack of a skull roof also means that these fish are likely amenable to both LSFM and confocal microscopy for shallow to mid-range depths, though neither technique has been thoroughly explored in *Danionella* species. It is unknown how well these animals tolerate long-term imaging, or whether they could be successfully intubated for long-term experiments. The ability to image fast events such as calcium dynamics, as well as longer-term events such as neurodegenerative processes, glia-neuron interactions, or neuronal development would increase their attractiveness as a model. Though additional work remains to establish protocols and best practices for imaging in *Danionella* species, the ease of imaging *Danionella* at both the larval and adult stages enables previously impossible or impractical longitudinal studies of development, aging, and neurological disease. This underscores their importance as an emerging vertebrate model.

#### Zebra Finch

##### Background

A well-established animal model with largely unexplored imaging potential is the zebra finch, *T. guttata*. Zebra finches are a songbird species from Australia that are easy to breed and reach sexual maturity in 70–80 days, making them one of the fastest maturing bird species. The greatest potential of the zebra finch for modeling neurobiology is its song: Finches and other songbirds reflect human speech patterns in their capacity to learn, recall, and perform complex songs. The circuits directing songbird vocalization have been used to investigate the neurobiology behind practice and performance, sexual differences in vocal learning, motor control of speech, and the interplay between motor control and vocal learning.

##### Behavior

Juvenile male finches learn their song from a male tutor, typically the father, and their vocal learning shares much in common with human vocal learning. The subsong produced by young zebra finch parallels human infant “babbling,” where both generate random syllables to dial in the correct notes that will eventually form their adult song or speech (Aronov et al., 2008). The adult song is highly stereotyped, though differences between individuals exist, making it easy to quantify changes in song pattern (Mello, 2014).

Many neurological diseases such as Huntington’s, Parkinson’s, and Fragile X syndrome result in changes in speech. Yet, the underlying mechanisms behind loss of normal speech patterns in neurological disease cannot be truly mirrored by any of the dominant research organisms outside of zebra finch. Rodents can vocalize, including audible squeaks and ultrasonic vocalizations (USVs), but are not known to imitate sounds or learn vocalizations. This can lead to unclear results when studying genes known to affect vocal learning, such as *Foxp2*. Global *Foxp2* knockdown has been accomplished in mouse, and area-specific *Foxp2* knockdown has been performed in mouse and zebra finch (Haesler et al., 2007; French and Fisher, 2014; Norton et al., 2019; Urbanus et al., 2020). In mice, results for whether USVs were affected in *Foxp2* global heterozygous knockout pups was inconsistent, and region-specific knockouts in the cortex, striatum, or cerebellum did not produce a deficit in USVs (French and Fisher, 2014; Urbanus et al., 2020). In zebra finch, region-specific knockdown of *Foxp2* using lentiviral shRNA in a region known to be important for song production (Area X) impaired the ability of young male finches to copy their tutor’s song (Haesler et al., 2007). This defect in song learning reflects the developmental vocal dyspraxia shown by humans with mutation of FoxP2 (Lai et al., 2001).

##### Genetics and Disease Models

Currently, the relative lack of genetic tools in birds has been a hinderance for their use as models of neurological disease. Great efforts have been made to interrogate why traditional virus-mediated transfection and primordial germ cell (PGC) editing are more difficult in zebra finch compared to other birds like the chicken. Recent studies have shown that divergence in the low-density lipoprotein receptor of zebra finches is one of the factors behind inefficient lentiviral transfection, as it is the main receptor for the VSV G protein component of the lentiviral system (Velho et al., 2021). Significant differences exist between zebra finch and chicken PGCs, including different distribution and developmental gene-expression programs, which may further underly difficulties in transgenesis (Jung et al., 2019). Alternative strategies, such as electroporation and lentiviral injection into the brain have been successful at producing tissue-targeted zebra finch knockdowns and transgenics (Haesler et al., 2007; Ahmadiantehrani and London, 2017; Norton et al., 2019).

Germ line transgenesis of zebra finches have been achieved, but with low-efficiency (Agate et al., 2009; Abe et al., 2015; Liu et al., 2015). Recent reports have improved efficacy of transgenesis by direct lentiviral infection of cultured PGCs (Gessara et al., 2021), resulting in transgenic songbirds expressing eGFP in various tissues including neurons in the song circuits. CRISPR/Cas9 editing has been achieved in quail and chicken, and adenovirus-mediated CRISPR/Cas9 delivery has been used to edit zebra finch PGCs, but no adult mutant has yet been generated in zebra finch (Lee et al., 2019; Xu et al., 2020; Jung et al., 2021). Successful use of CRISPR/Cas9 in zebra finch would be a great leap forward in the tractability of this model for the study of neurodegenerative diseases.

Even with the current difficulty of transgenesis, a transgenic models for Huntington’s disease has been produced (Liu et al., 2015). Parkinson’s has also been modeled in the finch through reduction of presynaptic dopamine input to Area X of the finch brain using 6-hydroxydopamine injection (Miller et al., 2015). HD model finches had severe defects in song learning and adult song maintenance, as well as other pathology associated with human HD-like loss of striatal neurons and HTT protein aggregates (Liu et al., 2015). The dopamine-reduction PD model resulted in changes to normal vocal variability (Miller et al., 2015).

##### Microscopy in the Zebra Finch Model

Current studies in finch mostly rely on electrophysiology and examination of the song pattern to understand how changes in neuronal function led to changes in song. Most imaging studies in finches are done in fixed tissue using confocal or widefield detection, and fluorescent markers or antibodies. A host of validated antibodies and their localization in the brain can be found at the Zebra Finch Expression Brain Atlas.^2^ Recently, both expansion microscopy and LSFM have been used to image neurons and vasculature in the zebrafish brain, though use of LSFM in adult finch brains requires tissue clearing to reduce scattering. There remain some hurdles with tissue clearing in finch since existing clearing protocols have been primarily optimized for murine brain tissue. Rocha et al. (2019) report successful use of iDISCO+ and CUBIC clearing protocols in zebra finch adult brains and were able to visualize anatomical landmarks and the song systems of the finch *via* LSFM. Düring et al. (2019) have also used Expansion Microscopy to resolve individual neurons and their dendritic spines within the zebra finch brain using LSFM.

Fixed and sectioned tissues, rather than entire brains, still dominate studies of zebra finch song system projections. Though not a new technique in itself, organotypic brain slices have been recently used to image the finch brain at a higher resolution, including functional imaging using calcium sensors (Shen et al., 2017). The use of brain slices allows better access to tissues for specific labeling of structures and may prove useful for introducing other biosensors or looking at glial function. The initial use of biosensors and optogenetic tools has been successful in the zebra finch *in vivo.* Optogenetic control of song circuits using channel rhodopsin (hChR2-YFP) *in vivo* has been used to test mechanisms controlling song patterning and learning (Roberts et al., 2012). 2-photon microscopy with an installed cranial window has been used with head-fixed male zebra finches to record GECIs during female-directed song (Picardo et al., 2016), and to look at the differential response of GECI expressing high vocal center neurons and astrocytes during playback of the bird’s own song (Graber et al., 2013). Head-fixed 2-photon imaging has been successful in generating some higher-resolution data as well, notably to measure dendritic spine size in the high vocal center before and after a pupil bird listens to a tutor bird sing (Roberts et al., 2012). Further work to expand the toolkit for *in vivo* imaging with multi-photon and cranial window or thinned skull techniques would enhance the zebra finch’s potential as a major brain disease model. Additional ways to differentially label multiple cell types within the living zebra finch could also expand our understanding of how supporting cells like glia contribute to successful vocal learning and memory. Imaging at the level of cellular resolution can help to further refine our understanding projections within the song system, and how neurological disease processes can lead to changes in vocal learning and speech function.

### Other Emerging Models

*“For such a large number of problems there will be some animal of choice, or a few such animals, on which it can be most conveniently studied.”* Krogh et al. (1929)

The models listed above are a limited selection of useful models that have already demonstrated great promise for neurobiological disease research, but this list is not comprehensive and does not represent the full landscape of all established and emerging model systems in neuroscience. Invertebrate models will continue to be important players in understanding human disease due to their simpler nervous systems and their comparatively scaled-down number of genes and transcripts. C. elegans and Drosophila have been immensely productive models for neuroscience for decades (Kimble and Nüsslein-Volhard, 2022). Both animals have detailed maps of their nervous systems available, and well-understood circuits that can be optogenetically probed thanks to the ease of genetic manipulations in both species (White et al., 1986; Schroll et al., 2006; Zimmermann et al., 2009; Leifer et al., 2011; Inagaki et al., 2014; Shipley et al., 2014; Scheffer et al., 2020). Many fundamental neuronal genes have been discovered in *C. elegans* and *Drosophila*. In Drosophila, these include the important neurodevelopmental genes *hedgehog* and *notch*, as well as the first identified learning mutants *dunce* (cAMP phosphodiesterase) and *rutabaga* (adenylate cyclase) (Dudai et al., 1976; Lehmann et al., 1981; Jürgens et al., 1984; Livingstone et al., 1984; Wieschaus et al., 1984; Fuccillo et al., 2006; Louvi and Artavanis-Tsakonas, 2006; Le Gall et al., 2008; de Bivort et al., 2009; Bellen et al., 2010). *C. elegans* research led to the discovery of the axon guidance protein netrin (UNC-6 and its receptors UNC-5 and UNC-40) (Hedgecock et al., 1990; Leung-Hagesteijn et al., 1992; Leonardo et al., 1997), and the identification of the circuits controlling touch sensation (Chalfie et al., 1985) among other important work. This foundation of established knowledge of circuits and molecular machinery in the nervous system of *Drosophila* and *C. elegans* now allows for very precise questions to be asked about complex circuit function, learning and memory, and *Drosophila*’s smaller size allows for whole-brain live imaging during behavior (Lemon et al., 2015; Huang et al., 2018; Aimon et al., 2019; Mann et al., 2021).

Invertebrates have the potential to be much more than vessels for cell biological studies as they possess useful behavioral traits that can inform the study of genes in neurobiological disease. To continue to use classical invertebrates as an example: *C. elegans* sleep, react to attractive and repulsive cues, and search for food and mates and *Drosophila* perform elaborate courtship dances, show aggression to compete for resources, and are capable of olfactory and visual learning. Since *C. elegans* and *Drosophila* are both members of the same group, Ecydysozoa, an additional argument can be made for expanding into other invertebrate models to add additional breadth to comparative studies. Emerging invertebrate models include cephalopods, hydra, chordates, rotifers, and planaria. These models share a commonality of maintained regenerative capacity throughout life and have gained traction as potential models of aging and mechanisms of regeneration (Austad, 2009; Murthy and Ram, 2015; Voskoboynik and Weissman, 2015; Gribble and Mark Welch, 2017). Both aging and regeneration are of broad interest for neurodegenerative disease: to understand and prevent the risk of neurodegenerative disease associated with aging (Hou et al., 2019; Azam et al., 2021), and to help recover cognitive function in patients suffering from neurodegeneration.

There are also alternative vertebrate models of interest in aging and regeneration (Table 1). Since lifespan varies broadly among organisms, it is possible to select animals that age at different rates to investigate the impacts of slower or faster aging on neurobiological processes. The African turquoise killifish, *Notobranchius furzeri*, is a short-lived (∼4–8 months) vertebrate that reaches maturity in as little as 2 weeks and displays aging-associated disease phenotypes (Harel and Brunet, 2015; Russell et al., 2017; Vrtílek et al., 2018). Its lifespan is considerably shorter than other vertebrates used to study aging, such as mouse and zebrafish (3.5- and 4-year maximal lifespans, respectively), greatly accelerating the pace at which experiments can be performed.

Multiple vertebrates are capable of at least some level of regeneration or increased neuroplasticity. Ground squirrels and birds are known to go through seasonal remodeling of their brains. Ground squirrels reversibly remodel their retinas and associated synapses during winter torpor (Merriman et al., 2016) while food-storing birds show seasonal changes in hippocampus size and neuronal recruitment into the hippocampus (Sherry and MacDougall-Shackleton, 2015; Pozner et al., 2018). Amphibians like *Xenopus* and axolotls are also highly regenerative, however, unlike *Xenopus*, axolotls and other salamanders retain their neuroregenerative abilities after metamorphosis (Filoni et al., 1977; Bosco, 1979; Joven et al., 2019; Phipps et al., 2020). Comparative analysis of genes involved in regenerative processes across species, and between regenerative and non-regenerative species, will help us understand the pathways involved in regeneration and provide potential druggable targets and new therapeutics for neurodegenerative disease, stroke, and traumatic injury.

Many of the models discussed in this section have not yet been used in imaging studies. Building the reagents and protocols necessary for fixed tissue imaging, let alone live imaging, is non-trivial and not every model will be suited for microscopy approaches. However, successful strategies for tackling stubborn transgenesis or imaging in one model inform approaches for how to solve similar problems in subsequent models. For example, both axolotls and *Xenopus* contain the same three types of pigment as zebrafish (Masselink and Tanaka, 2021). Indeed, albino strains of both *Xenopus* and Axolotl already exist, but retain some pigmentation (Frost and Malacinski, 1979; Woodcock et al., 2017; Fukuzawa, 2021). Forward genetic screens to create pigmentless Axolotls would be impossible, but since pigment-producing genes have been identified and targeted in zebrafish, CRISPR/cas9 mutagenesis could be used to edit these genes in amphibians to produce transparent models more suited to imaging A *Xenopus* mutant lacking yellow pigment has been recently generated, and further targeting of known pigment-related genes could lead to the production of completely pigmentless amphibians (Nakajima et al., 2021).

## Discussion

Modeling neurodegenerative diseases largely falls into the categories of “modeling known components,” such as genes and pathologies, and “uncovering unknown etiologies of disease”–these could be genes, cells, tissues, or extracellular factors involved. The introduction of new model organisms into the field of neurodegenerative disease research could help answer questions in both categories. Comparative studies of how different species react to the pathological and genetic drivers of neurodegenerative disease can help us understand the pathways that mediate disease, discover new genes involved, and develop potential therapeutics.

For a model to be widely utilized, it must fill a required niche. The non-mammalian vertebrate models discussed in this review all complement existing mammalian research and fill in the gaps of what cannot be done in mammals or invertebrates. To succeed, the model must also be supported by an enthusiastic and invested community. For example, *C. elegans*, *Drosophila*, and zebrafish have all benefitted greatly from a strong community and wide sharing of resources among labs. It is essential for information on husbandry, basic protocols, genome sequence, and anatomical descriptions to be easily available and disseminated. Therefore model organism databases such as FlyBase, WormBase, and ZFIN serve as irreplaceable resources for researchers working on model organisms. Community advocacy is also an important factor in normalizing the use of new models and advocating for funding. New models often face steep challenges associated with obtaining funding and acceptance of a model’s relevance to a research topic.

To push neuroimaging forward in these emerging models, a solid base of genetic tools is needed. A method of genetic editing is needed to create disease-specific models and tag endogenous proteins. CRISPR/cas9 is generally successful in a variety of animals, but as demonstrated in zebra finch, the road to genetic editing is not always straightforward. Imaging of specific neurons or areas of the brain *in vivo* requires the development of techniques to generate transgenic animals and the identification of tissue-specific promotors to control expression of fluorescent proteins.

To visualize labeled tissues it is useful to generate animals devoid of pigment or develop protocols for installation of cranial windows. Cranial window are an area of active development with newly designed windows for rodent models that are flexible and easier to work with (Cramer et al., 2021). Still the use of animal models that do not require cranial windows is an important biological consideration given the concerns of wound healing responses to the window surgery itself. Imaging technology continues to evolve as well with new infrared laser sources and adaptive optics for imaging deep into specimens, computational optics-based systems that enable smart microscopy to image more selectively, and technical efforts that continue to push the spatial and temporal resolution of modern optical imaging. One important aspect seen in both modern imaging technology and animal model work is the key role of community. As discussed previously, community directly drives animal model research: the strong *Drosophila*, zebrafish, and *C. elegans* communities are great examples of this with their own genetic repositories, data websites, technical newsletters, and research conferences. These communities embrace open access science with free sharing of reagents, methods, and specimens. The imaging community is also rapidly embracing this concept of open access with not only well established open source software such as the widely adopted ImageJ but the increasing trend toward open hardware systems such as OpenSPIM (Pitrone et al., 2013). This open sharing of both the model itself and all the tools utilized not only helps in established models but will aid in the discovery and adoption of new research models of important neuroscience problems. Modern neuroscience needs a range of models and tools to solve its mysteries and open science greatly drives this. The tandem of imaging and animal models exemplifies this community approach, and this will continue to drive great innovation in both biological systems and the tools used to study them.

## Author Contributions

The project was first conceptualized by EMH. All authors wrote the manuscript, contributed to the article, and approved the submitted version.

## Conflict of Interest

The authors declare that the research was conducted in the absence of any commercial or financial relationships that could be construed as a potential conflict of interest.

## Publisher’s Note

All claims expressed in this article are solely those of the authors and do not necessarily represent those of their affiliated organizations, or those of the publisher, the editors and the reviewers. Any product that may be evaluated in this article, or claim that may be made by its manufacturer, is not guaranteed or endorsed by the publisher.

## Footnotes

1 Non-human primates considered include marmoset, macaque, squirrel monkey, vervet monkey, and mouse lemur. Other common models considered include: *C. elegans*, chick, Drosophila, zebrafish, Xenopus, and zebra finch. Results were generated from PubMed searches for the name of the model organism + “neurobiology”.

2
http://www.zebrafinchatlas.org/
